# Insomnia and the risk of depression: a meta-analysis of prospective cohort studies

**DOI:** 10.1186/s12888-016-1075-3

**Published:** 2016-11-05

**Authors:** Liqing Li, Chunmei Wu, Yong Gan, Xianguo Qu, Zuxun Lu

**Affiliations:** 1 School of Public Health, Tongji Medical College, Huazhong University of Science and Technology, No. 13 Hangkong Road, Wuhan, Hubei 430030 China; 2 School of Economics and Management, Jiangxi Science and Technology Normal University, Nanchang, Jiangxi China; 3 School of Basic Medicine, Gannan Medical University, Ganzhou, Jiangxi China; 4 School of Health Management, Hangzhou Normal University, Hangzhou, Zhejiang China

**Keywords:** Insomnia, Sleep disorders, Depression, Meta-analysis, Epidemiology

## Abstract

**Background:**

Observational studies suggest that insomnia might be associated with an increased risk of depression with inconsistent results. This study aimed at conducting a meta-analysis of prospective cohort studies to evaluate the association between insomnia and the risk of depression.

**Methods:**

Relevant cohort studies were comprehensively searched from the PubMed, Embase, Web of Science, and China National Knowledge Infrastructure databases (up to October 2014) and from the reference lists of retrieved articles. A random-effects model was used to calculate the pooled risk estimates and 95 % confidence intervals (CIs). The *I* ^*2*^ statistic was used to assess the heterogeneity and potential sources of heterogeneity were assessed with meta-regression. The potential publication bias was explored by using funnel plots, Egger’s test, and Duval and Tweedie trim-and-fill methods.

**Results:**

Thirty-four cohort studies involving 172,077 participants were included in this meta-analysis with an average follow-up period of 60.4 months (ranging from 3.5 to 408). Statistical analysis suggested a positive relationship between insomnia and depression, the pooled RR was 2.27 (95 % CI: 1.89–2.71), and a high heterogeneity was observed (*I* ^*2*^ = 92.6 %, *P* < 0.001). Visual inspection of the funnel plot revealed some asymmetry. The Egger’s test identified evidence of substantial publication bias (*P* <0.05), but correction for this bias using trim-and-fill method did not alter the combined risk estimates.

**Conclusions:**

This meta-analysis indicates that insomnia is significantly associated with an increased risk of depression, which has implications for the prevention of depression in non-depressed individuals with insomnia symptoms.

## Background

Depression is a common mental disorder and is described as a continuum ranging from a few depressive symptoms to major depression [1]. It is one of the leading global burdens of disease (GBD) and is estimated to be one of the top three health concerns by 2020 [2, 3]. Some evidence showed that 5.8 % of men and 9.5 % of women would experience a depressive episode in any given year for a lifetime [4]. Among older adults in Japan, depression is one of the most common diseases and is a leading cause of morbidity and mortality [5, 6].

Insomnia is the subjective feeling of having difficulties initiating or maintaining sleep (DIS and DMS respectively, jointly referred to as DIMS) or of non-restorative sleep (NRS) [7, 8]. Epidemiological studies have shown that 20 to 35 % of the general population report insomnia symptoms, and that 10 to 20 % have clinically significant insomnia syndrome [9–13]. Insomnia prevalence has been found to be associated with measurements of worse physical and mental health [14].

Both insomnia and depression are major public health problems. It has been reported that insomnia is associated with an increased risk of depression and/or anxiety disorders [9, 15]. The identification of modifiable risk factors for depression has a greatly important implication for the primary prevention. Many observational studies have focused on whether insomnia has an influence on depression risk [9, 16–23]. In 2011, Baglioni et al. [24] performed a meta-analysis to investigate the association between insomnia and the risk of depression, and the results showed that an overall odds ratio (OR) for insomnia to predict depression of 2.60 (95 % confidence interval (CI):1.98–3.42). Since then, many new observational studies have emerged, and some of them had large sample sizes and long follow-up lengths. In addition, the previous review only conducted a subgroup analysis by different age groups of participants. The incidences and the risk factors for depression might vary with the definitions of depression and the exposure changes, or vary in samples from different gender, follow-up durations, and geographic regions. Including more studies and enlarging the sample size would be important for strengthening the reliability of describing the association between insomnia and depression risk. Therefore, we conducted an updated-analysis to further investigate the issue.

## Methods

### Search strategy

This meta-analysis was performed according to the checklist of the Meta-analysis Of Observational Studies in Epidemiology (MOOSE) guidelines [25]. The systematic literature search was conducted by two investigators (L.Q.L. and C.M.W.) independently through the PubMed, Embase, Web of Science and China National Knowledge Infrastructure (CNKI) databases for pertinent studies published in English and Chinese from their inception to October 2014. The key words used as the search terms were the following: “insomnia”, “sleep disorder”, “sleep disturbance”, “sleep problem”, “sleep quality”,”sleep duration” in combination with “depression”, “mental disorder”, and “anxiety”. The search was restricted to studies in humans. In addition, the reference lists of all identified relevant publications were reviewed.

### Inclusion criteria and exclusion criteria

The eligibility of each study was assessed independently by two investigators (L.Q.L. and C.M.W.), and disagreements were resolved through consultation with the third investigator (Z.X.L.). Studies meeting the following criteria were included in the meta-analysis: (1) the main exposure of interest was insomnia and the outcome of interest was depression; (2) the study design was prospective cohort; (3) insomnia was characterized by DIS and/or DMS or NRS; (4) depression was measured by self-reported symptom scales, physician/clinician diagnosis, or structured clinical diagnostic interview [24]; and (5) the study reported a ratio-based measurement of association of insomnia with depression.

Studies were excluded if: (1) the study was not published as the full reports, such as case reports, commentaries, conference abstracts and letters to editors; (2) the study had a retrospective design; (3) participants with depression at baseline were not excluded for the analysis or the effect of symptoms of insomnia on predicting depression was not controlled for other depressive symptoms at baseline [24]; and (4) Both insomnia and depression acted as the exposure resulting in predicting the other disorder (such as anxiety). If duplicate publications from the same study were identified, we would include the result with the largest number of individuals from the study.

### Data extraction

The following information was extracted for each study: name of the first author, publication year, study name, source of the participants, geographic region, gender, mean age of the participants at baseline, insomnia measurement, definition of insomnia based on DSM-IV-TR criteria [8], depression measurement, length of the follow-up period, number of the follow-up assessments, sample size, the OR, relative risk (RR) or hazard ratio (HR) with 95 % CI, and covariates that were adjusted in the multivariable analysis.

### Quality assessment

Two investigators (C.M.W. and Y.G.) independently fulfilled the quality assessment using the Newcastle-Ottawa Scale [26], which is a validated scale for non-randomized studies in meta-analysis. The Newcastle-Ottawa Scale is a nine-point scale that allocates points on the basis of the process of selection of the cohort study and measurement of exposure (0–4 points), the comparability of cohorts (0–2 points) and the identification of the outcome and adequacy of follow-up (0–3 points). We assigned scores of 0–3, 4–6, and 7–9 for the low, moderate, and high quality of studies, respectively.

### Statistical analysis

The RR was considered as the common measure of the association between insomnia and depression. The HR was considered to be equivalent to RR, and the OR was transformed into the RR. OR was corrected according to the following formula: $\mathrm{R}\mathrm{R}=\frac{OR}{\left(1-{P}_0\right)+\left({P}_0\times OR\right)}$ [27]. In the cohort study, *P*_*0*_ indicated the incidence of the outcome of interest in the non-exposed group. We preferentially pooled multivariable adjusted risk estimates where such estimates were reported. Where adjusted analysis was not available, we pooled the unadjusted estimates. The RRs for the associations between insomnia and the risks of depression were pooled using the fixed-effects model where heterogeneity was not detected, or the random-effects model was used otherwise.

For further confirmation and assessment of the association between insomnia and the risk of depression, subgroup analysis was carried out to explore the sources of potential heterogeneity and examine the robustness of the primary results. The differences among subgroups were tested by meta-regression analysis (using STATA ‘metareg’ command). In sensitivity analysis, we conducted a leave-one-out analysis [28] to observe the magnitude of influence of each study on the pooled RR.

Statistical heterogeneity among studies was evaluated with the *Q* and *I* ^2^ statistics. For the *Q* statistic, statistical significance was set at *P* < 0.1 and for the *I* ^2^, the values of 25 %, 50 % and 75 % respectively denoted cut-off points for low, moderate and high degrees of heterogeneity [29]. Potential publication bias was evaluated with a funnel plot and the Egger’s test [30]. The Duval and Tweedie nonparametric trim-and-fill methods [31] were performed to further assess the potential publication bias. All statistical analyses were performed with STATA statistical software (version 12.0; College Station, TX, USA). All reported probabilities (*P* values) were two-sided, with a significance level of 0.05 except where otherwise specified.

## Results

### Literature search

**Fig. 1:**
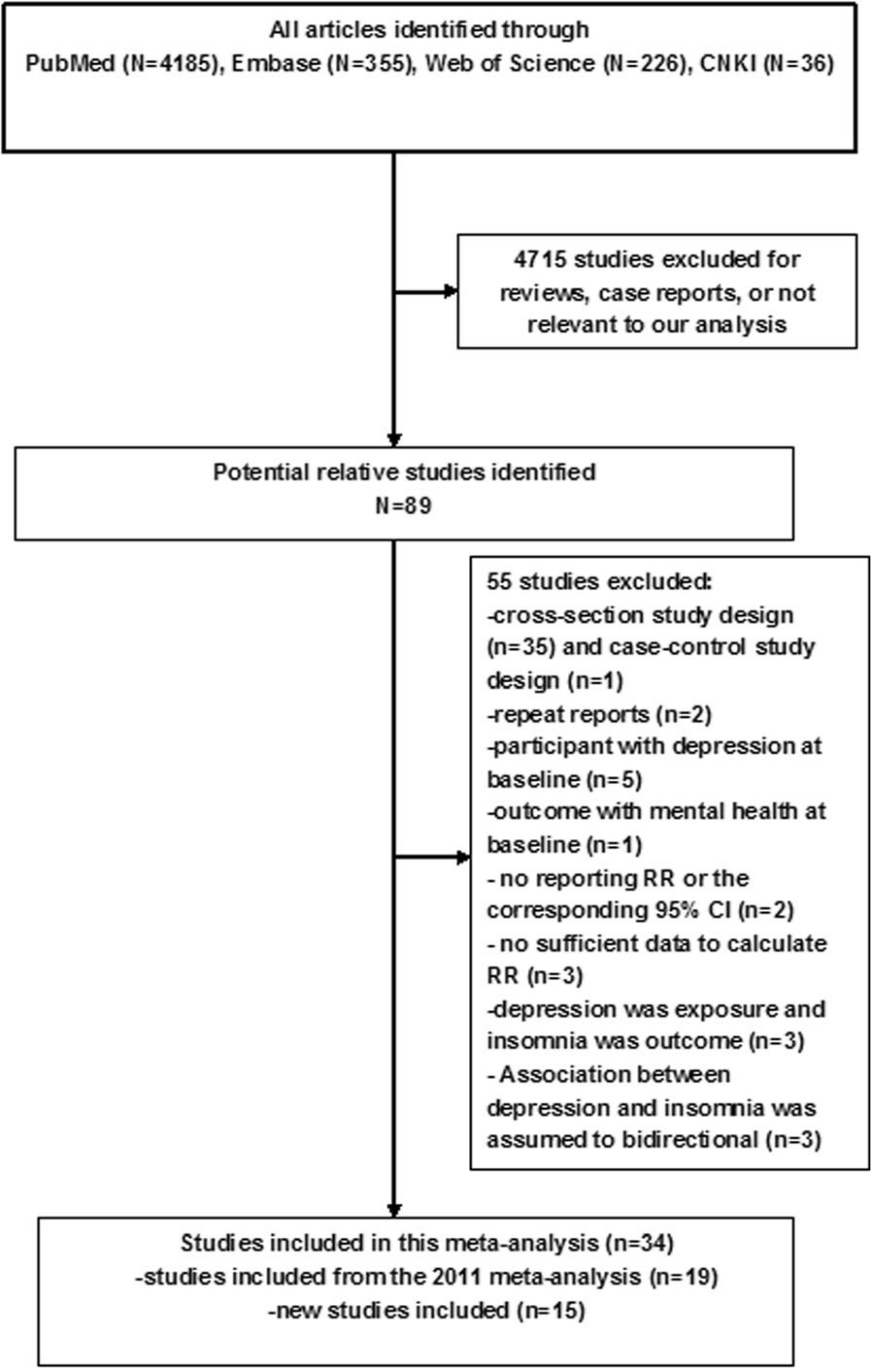
Flow chart of study selection of insomnia in relation to depression

Figure 1 presents the process of this study selection. The search strategy identified 4,802 articles, in which 4,185 articles from the PubMed, 355 articles from the Embase, 226 from the Web of Science, and 36 from the CNKI were retrieved. Of these, based on abstracts or titles, the majorities were excluded after the first screening because they were reviews, case reports, or not relevant to our analysis. After full-text review of the remaining 89 studies, 55 studies were excluded for the reasons shown in Fig. 1. Of note, all of the 21 studies included in the previous review were eligible according to the criteria in our research except two duplicated studies [7, 16] used the same samples as the other two studies [20, 32], and articles with longest follow-up and more detailed information were retained. Thus, 34 eligible cohort studies were finally included in this meta-analysis.

### Characteristics of studies

The included 34 studies [9, 10, 17–23, 32–56] were published between 1989 [9] and 2014 [32, 55, 56], and characteristics of them are shown in Table 1. The sample sizes of the cohorts ranged from 147 [40] to 44,270 [56] with a total of 172,077. The length of follow-up duration ranged from 3.5 [32] to 408 [18] months in this study, with an average of 60.4 months. Fourteen studies were conducted in the United States [9, 10, 17–19, 22, 23, 34–36, 38, 40, 51, 52] and in Europe [20, 32, 33, 37, 39, 41, 42, 45–47, 49, 50, 52, 53, 56] respectively, two studies in Australia [44, 55], and four studies in Asia [21, 43, 48, 54]. Three studies reported results for males only [18, 44, 52], and three studies reported results for females only [32, 46, 55], 27 studies reported results for both males and females [9, 10, 17, 19–23, 33–43, 45, 47–51, 53, 54, 56],and one study [47] reported results for males and females separately. In the present study, 11 studies [9, 10, 19–21, 32–34, 36, 38, 42, 46, 48, 52, 56] diagnosed insomnia on the basis of all DSM- IV criteria [57]: sleep difficulties, duration and daytime consequence (sd, dur and day), 10 studies [10, 23, 33, 34, 36, 47, 50, 51, 53, 54] based the diagnosis only on sleep difficulties and duration criteria (sd and dur), and 13 studies [17, 18, 22, 35, 37, 39, 40, 43–45, 49, 55] took into consideration only the sleep difficulties criterion (sd) [24]. Ten studies [18–20, 32, 45, 50, 51, 54, 55, 58] assessed insomnia repeatedly during the course of the follow-up, and the rest of the studies assessed insomnia at baseline. Interobserver agreement (κ) between two investigators was 0.98. The results of quality assessment are shown in Table 2. The average score for the quality assessment of included cohort studies was 6.6 (of a possible 9 points), which indicated that the quality for all studies was moderate-high in a whole.

**Table 1:** Characteristics of included studies in the meta-analysis

Study source	Study name	Sex	Insomnia measurement	DSM-IV-TR insomnia criteria satisfied	Depression measurement	Follow-up time (months)	Number of follow-up assessments	Baseline age (years)	No of participants	Covariates
Ford and Kamerow, 1989, USA [9]	National Institute of Mental Health Epidemiologic Catchment Area (ECA) study	M/F	Diagnostic Interview Schedule	sd^§^, dur^†^ and day	Diagnostic Interview Schedule	12	1	Range 18+; mean 45.79	7,954	Age, sex, socioeconomic status, race, and marital status
Brabbins et al., 1993, UK [33]	None	M/F	Geriatric Mental State	sd and dur	Geriatric Mental State	36	1	Range 65+; mean 69.76	701	No covariate adjustment
Breslau et al., 1996, USA [34]	None	M/F	NIHM Diagnostic Interview Schedule	sd and dur	NIHM Diagnostic Interview Schedule	42	1	Range 21–30; mean 26.14	979	Sex, hypersomnia, and number of other depressive symptoms
Chang et al. 1997 USA [18]	Johns Hopkins Precursors Study	M	Habit Survey Questionnaire	sd	Checklists medical reports and self-reports	408	7	Range NA; mean 26.3	941	Age at graduation, class year, parental history of clinical depression, coffee consumption, and measures of temperament
Weissman et al. 1997 USA [10]	National Institute of Mental Health Epidemiologic Catchment Area Study (ECA)	M/F	Questionnaires	sd and dur	Diagnostic Interview Schedule (DIS)	12	1	Range 18+; mean 48.23	7,113	Age, sex, and site
Foley et al. 1999 USA [35]	None	M/F	Interview	sd	CES-D	36	3	Range 65+; mean 80.09	6,899	No covariate adjustment
Johnson et al. 2000 USA [36]	None	M/F	1 item from the CBCL	sd and dur	CBCL and TRF	60	1	Range NA; mean 6	717	Sex, birth weight, and mother’s history of MDD using generalized estimation equations
Mallon et al. 2000 Sweden [37]	County of Dalarna registry	M/F	Uppsala Sleep Inventory	sd	HADS	144	1	Range 45–65; mean 55	1,244	Smoking, depression, and insomnia
Roberts et al. 2000 USA [17]	Alameda Country study	M/F	2 Items from the DSM-12D	sd	12 Items from the DSM-12D	12	1	Range 50+; mean 64.9	2,370	Age, sex, marital status, social isolation, education, financial problems, problems with daily activities, and heave drinking
Roberts et al. 2002 USA [38]	None	M/F	Questionnaires	sd, dur and day	Diagnostic Interview Schedule for Children	12	1	Range 11–17; mean 15	3,136	Age, values of the functioning measures at baseline, sex, parental education, and insomnia level
Hein et al. 2003 Germany [39]	None	M/F	Composite International Diagnostic Interview	sd	Composite International Diagnostic Interview	60	1	Range 55+; mean 60	664	No covariate adjustment
Perlis et al. 2006 USA [40]	None	M/F	HAMD (sleep items)	sd	SCID and HAMD	12	1	Range 60+; mean 72	147	No covariate adjustment
Morphy et al. 2007 UK [41]	None	M/F	Jenkins Sleep Scale	sd	HADS	12	1	Range 18+; mean 50	1,589	Age, sex, social class, anxiety (except when anxiety is the problem of interest), depression (except when depression is the problem of interest), and pain areas (except when widespread pain is the problem of interest)
Buysse et al. 2008 Switzerland [20]	The Zurich Study	M/F	SPIKE and visual analogue scales	sd, dur and day	SPIKE	240	6	Range NA; mean 19.5	278	Concurrent MDE at the time of insomnia diagnosis
Cho et al. 2008 USA [19]	Depression Substudy	M/F	PSQI	sd, dur and day	SCID and BDI	24	3	Range 60+; mean 69	329	Group status, depression symptoms, medical disease, age, sex, marital status, and education
Jansson-Fröjmark and Lindblom.2008 Sweden [42]	None	M/F	Basic Nordic Sleep Questionnaires and Uppsala Sleep Inventory	sd, dur and day	HADS	12	1	Range 20–60; mean 41.3	1,489	Age, sex
Roane and Taylor. 2008 USA [23]	National Longitudinal Study of Adolescent Health (Add Health)	M/F	In-home interview	sd and dur	In-home interview	78	1	Range 12–18; mean 16	3,582	Sex
Kim et al. 2009 Korea [21]	Kwangju community study	M/F	Interview	sd, dur and day	Geriatric Mental State	24	1	Range 65+; mean 72.2	792	Age, sex, education, housing, past occupation, current employment, living area, life events, social deficit, physical activity, GMS organicity, GMS anxiety, and daily drinking.
Szklo-Coxe et al. 2010 USA [22]	Wisconsin Sleep Cohort Study	M/F	PSG + interview and self-reported symptoms	sd	Zung Self-Rating Depression Scale	44	1	Range 33–71; mean 53.6	555	Age, sex, chronic health conditions, alcohol consumption, cigarette smoking, caffeine consumption, use of hypnotic agents, and BMI
Yokoyama et al. 2010 Japan [43]	Nihon University Japanese Longitudinal Study of Aging: (NUJLSOA)	M/F		Questionnaire	11-item short form of the CES-D	36	1	Range 69+; mean 73.1	3,065	Age, sex, educational history, place of residence, sleep duration, excessive daytime sleepiness, discomfort feeling in the legs, subjective sleep sufficiency, psychological stress, self-rated health, and activities of daily living
Jaussent et al. 2011 France [45]	French Three-City Study	M/F	Questionnaire and clinical interview	sd	CES-D	48	2	Range 65+; mean ≥ 60	3,824	Age, center, CES-D baseline, sex, education, living alone, coffee consumption, alcohol consumption, smoking, chronic disease, past major depression, disability, prescribed sleep medication intake, and homeopathic and non-prescription treatments for sleep
Almeida et al. 2011 Australia [44]	Health In Men Study (HIMS)	M	Questionnaire	sd	Medical records (based on ICD-10)	72	1	Range 70+; mean ≥ 60	5,127	Age, education group, migrant status, living alone, low social support group, smoking group, BMI, diabetes, hypertension, arthritis, chronic respiratory diseases, coronary artery disease, stroke, and cancer
Marques et al. 2011 Portugal [46]	None	F	Interview	sd, dur and day	DSM-IV	5	1	Range 18–44; mean 29.8	382	Negative affect, positive affect, and lifetime depression
Thomée et al. 2011 Sweden [47]	None	M/F	A single item adapted from the Karolinska Sleep Questionnaire	sd and dur	Two items from the Primary Care Evaluation of Mental Disorders (Prime-MD)	12	1	Range 20–24; mean < 60	1,455	Relationship status, educational level, and occupation
Okajima et al. 2012 Japan [48]	None	M/F	PSQI	sd, dur and day	CES-D	24	1	Range 20+; mean 57.4	1,577	Age, sex, disease currently treated, habitual alcohol ingestion, smoking habit, and living alone
Salo et al. 2012 Finland [49]	Finnish Public Sector Study	M/F	The 4-item Jenkins Sleep Problem Scale	sd	Health register and medical records	42	1	Range 19–70; mean 43.9	40,791	Age, sex, socioeconomic position, night/shift work, health behaviors, baseline physical health, use of pain killers, psychological distress, and anxiety
Skapinakis et al. 2013 UK [53]	UK National Psychiatric Morbidity survey	M/F	The Revised Clinical Interview Schedule (CIS-R)	sd and dur	The Revised Clinical Interview Schedule (CIS-R)	18	1	Range 16–74; mean 44.75	2,406	Age, sex, marital status, educational qualifications,occupational class, employment status, and other psychological symptoms
Gehrman et al. 2013 USA [51]	Millennium Cohort Study (MCS)	M/F	2 items from PHQ and PCL-C, and questionnaires	sd and dur	PHQ-9	84	2	Range NA; mean 33.1	8,902	Sleep duration, birth year, sex, race/ethnicity, educational level, and marital status
Suh et al. 2013 Korea [54]	Korean Genome and Epidemiology Study (KoGES)	M/F	Questionnaire	sd and dur	BDI	72	3	Range 43–73; mean 52.3	1,089	Age, sex, education level, employment status, marital status, physical health, smoking status, heavy drinking, and time interactions with each covariate
Paudel et al. 2013 USA [52]	Prospective Osteoporotic Fractures in Men (MrOS) Study	M	PSQI	sd, dur and day	Geriatric Depression Scale (GDS)	40.8	1	Range 67+; mean 75.9	2,352	Age, clinic site, baseline GDS score, health status, education, use of benzodiazepines, alcohol consumption, cognitive function, walks for exercise, impairments in activities of daily living and certain medical conditions
Campbell et al. 2013 UK [50]	North Staffordshire Osteoarthritis Project (NorStOP)	M/F	Jenkins Sleep Questionnaire	sd and dur	HADS	72	2	Range 50+; mean ≥ 60	2,373	Age, sex, marital status, employment status, alcohol intake, smoking status, and BMI
Jackson et al. 2014 Australia [55]	Australian Longitudinal Study on Women’s Health, (ALSWH)	F	Questionnaire	None (sleeping difficulties in the last 12 months)	Questionnaire	108	3	Range 22–27; mean < 60	5,702	Education level, body weight dissatisfaction, history of abuse, and binge drinking in 2000
Sivertsen et al. 2014 Norway [56]	Nord-Trøndelag health study HUNT Study (HUNT2 and HUNT3)	M/F	Questionnaire	sd, dur and day	HADS	132	1	Range 19–67; mean 45.3 (HUNT2) Range 20–89; mean 56.1 (HUNT3)	44,270	Age, sex, education, angina, arthrosis, asthma, ankylosing spondylitis, cancer, diabetes, fibromyalgia, headache, hypertension, myocardial infarction, obesity, osteoporosis, rheumatoid arthritis, stroke and whiplash at baseline.
Dørheim et al. 2014 Norway [32]	Akershus Birth Cohort	F	BIS	sd, dur and day	EPDS	3.5	2	Range 17.4–47.5; mean 31.5	2,088	No covariate adjustment

*Abbreviations*: *NA* not available, *F* female, *M* male, *NIHM* National Institute of Mental Health, *CES-D* Centre for Epidemiologic Studies Depression, *CBCL* Child Behavior Checklist, *EPDS* Edinburgh Postnatal Depression Scale, *GMS* Geriatric Mental State diagnostic schedule, *TRF* Teacher Report Form, *HADS* Hospital Anxiety and Depression Scale, *DSM-12D* 12-item scale for DSM depression, *HAMD* Hamilton Rating Scale for Depression, *SCID* Structured Clinical Interview for DSM Disorders, *SPIKE* Structured Psychopathological Interview and Rating of Social Consequences of Psychic Disturbances for Epidemiology, *PSQI* Pittsburgh Sleep Quality Index, *BDI* Beck Depression Inventory, *BIS* The Bergen Insomnia Scale, *PSG* polysomnographic assessment, *day* daytime consequences criterion

^§^sd, sleep difficulties criterion

^†^dur, duration criterion

**Table 2:** Quality assessment of studies^a^

Study source	Selection				Comparability	Exposure			Total score
	Representativeness of the exposed cohort	Selection of the non- exposed cohort	Ascertainment of exposure	Demonstration that outcome of interest was not present at start of study	Comparability of cohorts on the basis of the design or analysis	Assessment of outcome	Was follow-up long enough for outcomes to occur (> = 5 years)	Adequacy of follow up of cohorts (>80 %)	
Ford and Kamerow [9]	1	1	1	1	1	1	0	0	6
Brabbins et al. [33]	1	1	1	1	0	1	0	0	5
Breslau et al. [34]	1	1	1	1	1	1	0	1	7
Chang et al. [18]	1	1	1	1	2	1	1	1	9
Weissman et al. [10]	1	1	1	1	1	1	0	0	6
Foley et al. [35]	1	1	1	1	0	1	0	0	5
Johnson et al. [36]	1	1	0	1	1	1	1	1	7
Mallon et al. [37]	1	1	0	1	1	1	1	0	6
Roberts et al. [17]	1	1	0	1	0	1	0	1	5
Roberts et al. [38]	1	1	0	1	2	1	0	0	6
Hein et al. [39]	0	1	1	1	0	1	1	1	6
Perlis et al. [40]	0	1	1	1	0	1	0	0	4
Morphy et al. [41]	1	1	1	1	2	1	0	0	7
Buysse et al. [20]	1	1	1	1	1	1	1	0	7
Cho et al. [19]	1	1	1	1	2	1	0	1	8
Jansson-Fröjmark and Lindblom [42]	1	1	1	1	1	1	0	1	7
Roane and Taylor [23]	1	1	1	1	0	1	1	0	5
Kim et al. [21]	1	1	1	1	2	1	0	0	7
Szklo-Coxe et al. [22]	1	1	1	1	1	1	0	0	6
Yokoyama et al. [43]	1	1	1	1	2	1	0	0	7
Jaussent et al. [45]	1	1	1	1	2	1	0	0	7
Almeida et al. [44]	1	1	1	1	2	1	1	0	8
Marques et al. [46]	0	1	0	1	1	1	0	0	4
Skapinakis et al. [53]	1	1	1	1	2	1	0	0	7
Salo et al. [49]	1	1	1	1	2	1	0	1	8
Suh et al. [54]	1	1	1	1	2	1	1	1	9
Paudel et al. [52]	1	1	1	1	2	1	0	0	7
Campbell et al. [50]	1	1	1	1	2	1	1	1	9
Jackson et al. [55]	1	1	1	1	1	1	1	1	8
Sivertsen et al. [56]	1	1	1	1	2	1	1	1	9
Okajima et al. [48]	1	1	1	1	1	1	0	0	6
Thomée et al. [47]	0	1	1	1	1	1	0	0	5
Gehrman et al. [51]	0	1	1	1	2	1	1	0	7
Dørheim et al. [32]	0	1	1	0	0	1	0	0	3

^a^The study quality was assessed according to the Newcastle Ottawa Quality assessment scale for cohort studies. This scale awards a maximum of 9 points to each study: 4 for selection, 2 for comparability, and 3 for assessment of outcomes (for cohort study). 1 = “Yes”, 0 = “No”, “Unable to determine” or “Not available”

### Quantitative synthesis

**Fig. 2:**
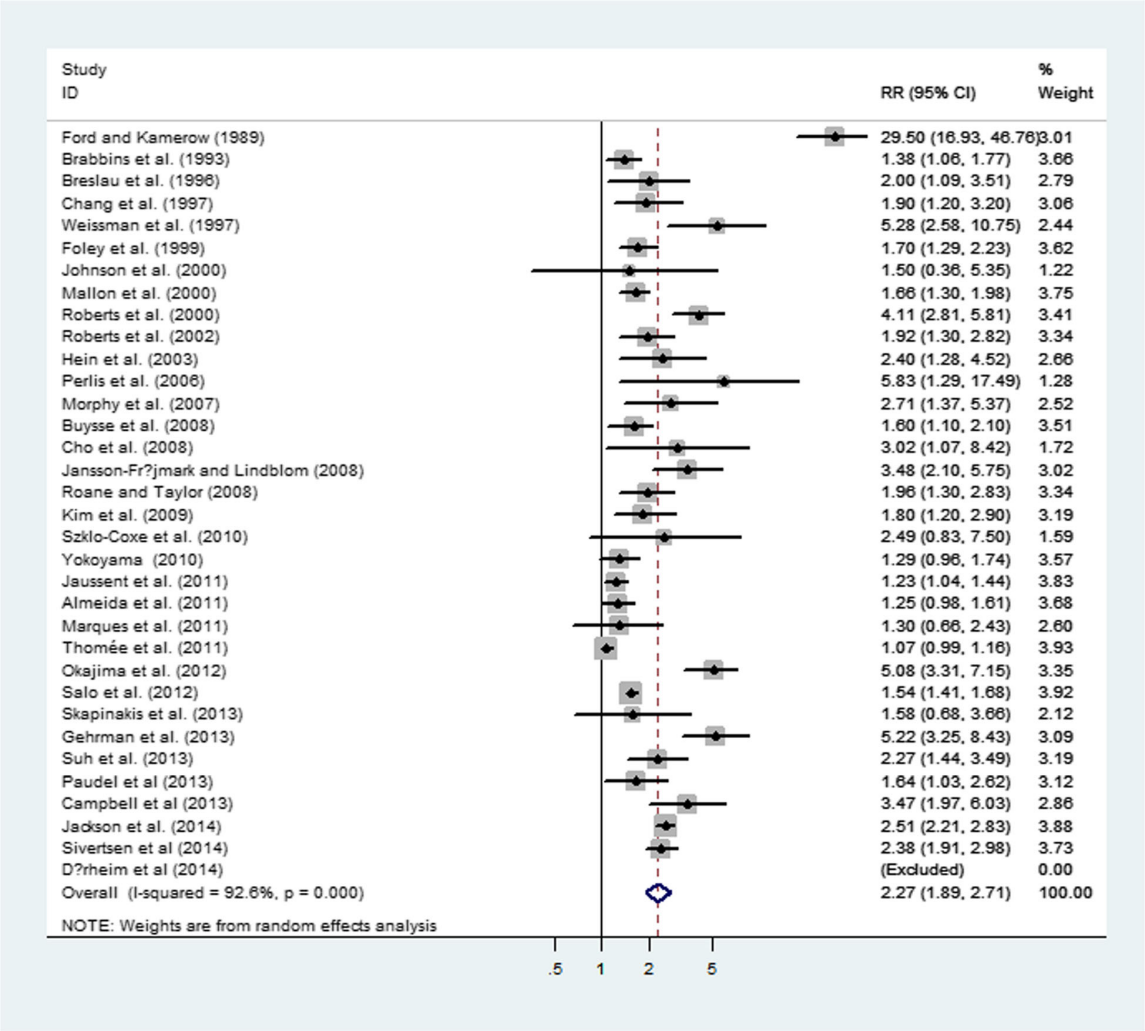
Forest plot of the association between insomnia and risk of depression

The results from the random-effects model combining the RRs for depression in relation to insomnia are shown in Fig. 2. Twenty-six studies suggested a significant positive relationship between insomnia and depression, while the other studies did not. The pooled RR of depression was 2.27 (95 % CI: 1.89–2.71) among populations with insomnia, and a high heterogeneity was observed among studies (*I* ^2^ = 92.6 %, *P* < 0.001).

### Subgroup analysis

Subgroup analysis was conducted by mean age at baseline, sex, study location, insomnia definition, type of depression measurement, follow-up duration, sample size, study quality, publication year (before 2010 vs. after 2010), and whether age, socioeconomic status, smoking status, alcohol intake, body mass index (BMI) were controlled or not in models. Insomnia was significantly associated with an increased risk of depression in all subgroups, with the exception of populations from Australia (RR = 1.79, 95 % CI = 0.90–3.54, *I* ^*2*^ = 95.9 %, *P* < 0.001). However, moderate to high heterogeneities were observed. No interactions between insomnia and stratification variables in relation to depression risk were observed (all *P* values for interactions > 0.05; Table 3).

**Table 3:** Subgroup analysis of relative risks for the association between insomnia and depression

	No of studies	RR (95 % CI)	*I* ^2^ (%)	*P* value for heterogeneity	*P* value between groups
Mean age at baseline, y					
<60	22	2.50 (1.96, 3.20)	94.70	<0.001	0.31
≥60	12	1.87 (1.47, 2.37)	80.70	<0.001	
Sex					
Male	3	1.46 (1.13, 1.88)	26.70	0.256	0.48
Female	3	1.96 (1.05, 3.66)	73.50	<0.001	
Mixed	28	2.41 (1.97, .95)	92.80	<0.001	
Study location					
USA	14	3.13 (2.03, 4.85)	89.80	<0.001	0.17
Europe	14	1.73 (1.43, 2.09)	88.50	<0.001	
Asia	4	2.27 (1.22, 4.21)	90.40	<0.001	
Australia	2	1.79 (0.90, 3.54)	95.90	<0.001	
Source of participants﻿*﻿					
General population	25	2.34 (1.85, 2.96)	94.00	<0.001	0.75
Non-general population	9	2.05 (1.53, 2.74)	78.70	<0.001	
Insomnia definition					
Sd, dur and day	11	2.90 (1.78, 4.74)	92.70	<0.001	0.37
Sd and dur	10	2.21 (1.50, 3.25)	90.30	<0.001	
Sd	13	1.87 (1.53, 2.29)	87.70	<0.001	
Type of depression measure					
Self-reported scales	17	2.20 (1.70, 2.86)	94.10	<0.001	0.63
Physician diagnosis	15	2.27 (1.67, 3.09)	91.80	<0.001	
Combined	2	3.89 (1.73, 8.74)	0.00	0.438	
Follow-up duration, y					
<5	22	2.34 (1.84, 2.97)	93.60	<0.001	0.77
5–10	8	2.37 (1.73, 3.25)	82.30	<0.001	
>10	4	1.88 (1.52, 2.32)	54.60	0.086	
Sample size					
<5000	26	2.01 (1.66, 2.44)	86.50	<0.001	0.10
≥5000	8	3.12 (2.11, 4.62)	96.40	<0.001	
Study quality					
Score >7	8	2.06 (1.60, 2.63)	88.60	<0.001	0.70
Score ≤ 7	26	2.36 (1.83, 3.03)	93.00	<0.001	
Publication year					
Before 2010	19	2.63 (1.95, 3.55)	88.20	<0.001	0.21
2010–2014	15	1.94 (1.53, 2.45)	94.62	<0.001	
Statistical model					
Unadjusted	5	1.78 (1.29, 2.47)	56.00	0.078	0.75
Adjusted	29	2.31 (1.90, 2.81)	93.40	<0.001	
Controlling age in models					
Yes	21	2.64 (2.04, 3.42)	92.30	<0.001	0.09
No	13	1.75 (1.33, 2.31)	92.50	<0.001	
Controlling SES in models					
Yes	17	2.34 (1.81, 3.03)	95.60	<0.001	0.78
No	17	2.15 (1.74, 2.65)	71.60	<0.001	
Controlling smoking status in models					
Yes	7	2.10 (1.45, 3.06)	89.80	<0.001	0.75
No	27	2.32 (1.88, 2.87)	93.30	<0.001	
Controlling alcohol intake in models					
Yes	9	2.47 (1.75, 3.48)	90.90	<0.001	0.69
No	25	2.19 (1.78, 2.69)	92.00	<0.001	
Controlling BMI in models					
Yes	5	2.19 (1.57, 3.06)	85.30	<0.001	0.94
No	29	2.28 (1.87, 2.78)	92.30	<0.001	

*Abbreviations*: *BMI* body mass index, *dur* duration criterion, *day* daytime consequences criterion, *F* female, *M* male, *NA* not available, *SES* socioeconomic status, *Sd* sleep difficulties criterion

*Study population truly or somewhat representative of a community or population-based study defined as general population, and study population was sampled from a special population (such as population from a company, register patients, data from the health insurance company or health examination organization or pregnant), which defined as non-general populations

### Sensitivity analysis

Sensitivity analyses were used to identify the potential sources of heterogeneity in association between insomnia and the risk of depression. This helped to examine the influence of various exclusions on the combined RR and test the stability of the quantitative synthesis results. In the leave-one-out analysis by omitting one study in turn, the overall combined RR did not change substantially, with a range from 2.07 (95 % CI: 1.77–2.42) to 2.33 (95 % CI: 1.95–2.78), and *I* ^2^ varied from 89.6 to 89.9 %. This indicated that none of the individual studies significantly influenced the overall result. It was worth noting that, six studies [32, 38, 41, 49, 50, 54] defined insomnia as DIS and/or DMS or NRS, and the rest of studies defined insomnia as DIS and/or DMS. There is controversy as to whether individuals with NRS complaint share similar pathophysiologic mechanisms with the other nocturnal symptoms, such as DIS and DMS. Restricting the analysis to the 28 studies defined insomnia as DIS and/or DMS yielded a pooled RR of 2.30 (95 % CI, 1.84 to 2.87) after exclusion of 6 studies. Thus, our main results would not change even if these six studies were excluded.

### Publication bias

**Fig. 3:**
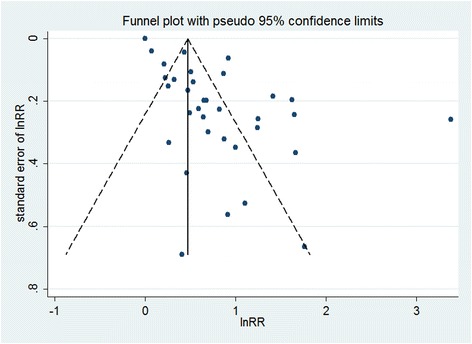
Funnel plot with pseudo 95 % confidence limits of insomnia and depression. The horizontal line represents the summary effect estimates, and the dotted lines are pseudo 95 % CIs

**Fig. 4:**
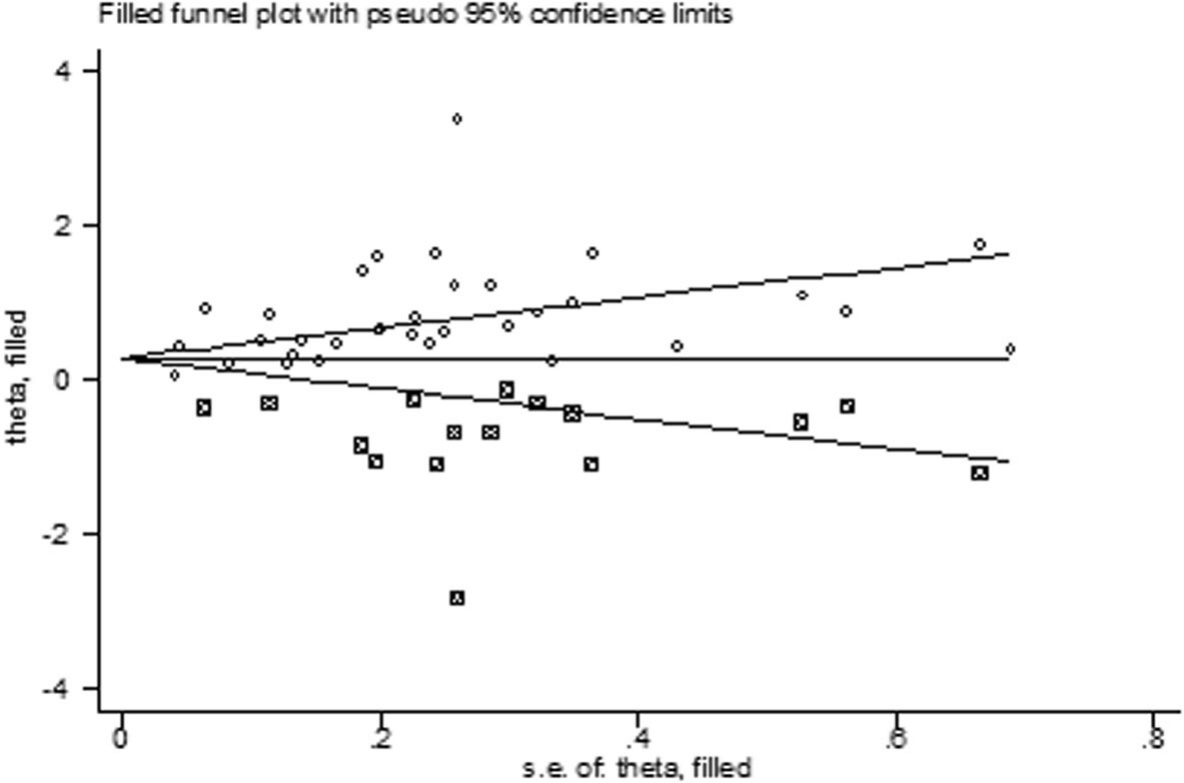
Filled funnel plot of RR from studies that investigated the association between insomnia and the risk of depression. The circles alone are real studies and the circles enclosed in boxes are ‘filled’ studies. The horizontal line represents the summary effect estimates, and the diagonal lines represent pseudo-95 % CI limits

The visual inspection of the funnel plot identified substantial asymmetry (Fig. 3). The Egger’s test identified evidence of substantial publication bias (*P* < 0.05). A sensitivity analysis using the trim-and-fill method was performed with 16 imputed studies, which produced a symmetrical funnel plot (Fig. 4). Using the trim-and-fill method, the RR was 1.40 (95 % CI, 1.16–1.69; *P* < 0.001). Correction for potential publication bias thus did not alter the significant association.

## Discussion

The results of this meta-analysis of 34 prospective cohort studies showed that insomnia was significantly associated with an increased risk of depression. The pooled estimates (RR = 2.27; 95 % CI: 1.89–2.71) indicated that participants with insomnia, compared to those free of it, experienced more than two-fold risk to develop depression. Furthermore, the association remained significant in most subgroup analyses.

### Comparison with previous study

Our findings were approximately consistent with those from the meta-analysis by Baglioni et al. in 2011 [24], which also showed that sleep difficulty was significantly associated with depression. The results of this current meta-analysis generally concur and further complement the findings of previous review in several important aspects. The present meta-analysis included 15 new prospective cohort studies with larger sample sizes and many more cases, which significantly enhanced the statistical power to detect potential association between insomnia and depression risk. Additionally, the previous review did not investigate any subgroups other than age. More importantly, compared with the previous review, the OR was corrected to more approximately the true RR in the present study, therefore our risk estimate is more accurate and a reliable. Of note, the associations differed among populations of different ethnic backgrounds were investigated in the present study. The current meta-analysis showed that the increased risk was more pronounced for participants from the United States than for European participants. However, no statistically significant association was observed in Australian populations, which might result from the limited number of included studies (two studies comprising 13,323 participants). In order to make the finding generalize to other populations, more studies are warranted to be conducted in other populations from Asia, Africa and South America.

There were several possible biological mechanisms through which insomnia in general may increase the risk of depression. Sleep disturbance may play a key role in the development of depression. Experimental studies showed that sleep loss may result in cognitive and affective alterations that lead to depression risk [55]. Alternatively, sleep disturbance impaired emotional regulation and stability [59] and may alter neural processes that may result in the symptomatology of depression [60]. Secondly, sustained arousal and chronic activation of hyperactivity of the hypothalamic–pituitary–adrenal (HPA) axis, the major neuroendocrine mediator of stress response, have been suggested as playing a vital role in the development of depression in insomniacs with objective short sleep duration [14, 61]. Finally, other proposed mechanisms by which insomnia might increase the risk of depression included increasing levels of inflammatory markers, such as C-reactive protein and interleukin-6 (IL-6) [62–64], which indicated low-level systemic inflammation was a predictor of depression development [65].

Long-term, double-blind, randomized controlled trials provided the best evidence on the effect between insomnia and depression. Recently, the study by Gosling et al. [66] showed that an internet-based insomnia intervention would indeed reduce the risk of depression. The role of insomnia treatment in modulating subsequent risk of depression needs to be studied further.

### Strengths and limitations

Our review is very valuable and crucial though it is an updated meta-analysis. First, our review added more than 3 times as many participants as the previous review, which provided stronger and more sufficient evidence. Second, the prospective nature of the included studies avoided the influence of recall and selection bias. Third, more studies from additional areas other than the North America and Europe were included, which increased the generalizability. Fourth, we did stratified analyses to explore whether the results were influenced by some confounding factors, and the consistent results from the sensitivity and subgroup analyses indicated that our findings were reliable and robust.

There are also some limitations in this meta-analysis. Firstly, the accuracy of our results might be influenced by the differences of the measurement criteria of insomnia and depression. However, no significant differences among groups were observed for the type of insomnia and depression measurement in this study. Secondly, we were unable to independently summarize the evidence of individual types of insomnia symptoms on depression risk due to no sufficient information in the original studies. Thirdly, although we extracted the most fully adjusted risk estimates, the adjusted confounders varied among the included studies. Some important confounding factors that might influence the relationship between insomnia and depression risk were gender, age, smoking, education, alcohol or drug abuse, other somatic or psychiatric disease, medication status, and social status. These important confounders were not fully adjusted in some of the included studies, which might influence the accuracy of the results. Finally, publication bias were detected, however, we used trim-and-fill method to correct the bias, which did not alter the significant positive association between insomnia and depression risk.

Based on our findings, we suggest that future research in this field is warranted, especially the long-term prospective cohort studies about the association between individual insomnia symptoms and depression. In addition, more interventional studies are necessary to explore the underlying mechanisms that link insomnia and depression.

## Conclusions

In conclusion, this meta-analysis supports the hypothesis that insomnia is associated with an increased risk of depression. Considering the increasing prevalence of insomnia worldwide and the heavy burdens of depression, the results of our study provide practical and valuable clues for the prevention of depression and the study of its etiology.

## Abbreviations

BMI: Body mass index
CI: Confidential interval
CNKI: China national knowledge infrastructure
DIS: Difficulties in initiating sleep
DMS: Difficulties in maintaining sleep
DSM-IV-TR: Diagnostic and statistical manual of mental disorders
GBD: Global burdens of disease
HPA: Hypothalamic–pituitary–adrenal
HR: Hazard ratio
IL-6: Interleukin-6
MOOSE: Meta-analysis of observational studies in epidemiology
NRS: Non-restorative sleep
OR: Odds ratio
RR: Relative risk.

## References

[1] Solomon A, Haaga DA, Arnow BA (2001). Is clinical depression distinct from subthreshold depressive symptoms? A review of the continuity issue in depression research. J Nerv Ment Dis.

[2] Murray CJ, Lopez AD (1997). Alternative projections of mortality and disability by cause 1990-2020: Global burden of disease study. Lancet.

[3] Murray CJL, Lopez AD. Global Burden of Disease: A comprehensive assessment of mortality and disability from diseases, injuries, and risk factors in 1990 and projected to 2020 summary. 1st edition. Cambridge: Harvard University Press; 1996.

[4] Organization WH (2001). The World health report: 2001: Mental health : new understanding, new hope.

[5] Services USDoHaH (1999). Mental health: a report of the surgeon general.

[6] Services USDoHaH. Report of the 2005 White House Conference on aging. The booming dynamics of aging: from awareness to action. Washington, DC; 2006. http://nicoa.org/wpcontent/uploads/2014/05/2005-WHCOA-Final-Report.pdf.

[7] Neckelmann D, Mykletun A, Dahl AA (2007). Chronic insomnia as a risk factor for developing anxiety and depression. Sleep.

[8] Association AP (2000). Diagnostic and Statistical Manual of Mental Disorders (DSM-IV-TR), 4th Edition, Text Revision.

[9] Ford DE, Kamerow DB (1989). Epidemiologic study of sleep disturbances and psychiatric disorders. An opportunity for prevention?. JAMA.

[10] Weissman MM, Greenwald S, Nino-Murcia G, Dement WC (1997). The morbidity of insomnia uncomplicated by psychiatric disorders. Gen Hosp Psychiatry.

[11] Ohayon MM (1997). Prevalence of DSM-IV diagnostic criteria of insomnia: distinguishing insomnia related to mental disorders from sleep disorders. J Psychiatr Res.

[12] Leger D, Guilleminault C, Dreyfus JP, Delahaye C, Paillard M (2000). Prevalence of insomnia in a survey of 12,778 adults in France. J Sleep Res.

[13] Ohayon MM (2002). Epidemiology of insomnia: what we know and what we still need to learn. Sleep Med Rev.

[14] Fernandez-Mendoza J, Vgontzas AN (2013). Insomnia and its impact on physical and mental health. Curr Psychiatry Rep.

[15] Gillin JC (1998). Are sleep disturbances risk factors for anxiety, depressive and addictive disorders?. Acta Psychiatr Scand Suppl.

[16] Vollrath M, Wicki W, Angst J (1989). The Zurich study. VIII. Insomnia: association with depression, anxiety, somatic syndromes, and course of insomnia. Eur Arch Psychiatry Neurol Sci.

[17] Roberts RE, Shema SJ, Kaplan GA, Strawbridge WJ (2000). Sleep complaints and depression in an aging cohort: A prospective perspective. Am J Psychiatry.

[18] Chang PP, Ford DE, Mead LA, Cooper-Patrick L, Klag MJ (1997). Insomnia in young men and subsequent depression. The Johns Hopkins precursors study. Am J Epidemiol.

[19] Cho HJ, Lavretsky H, Olmstead R, Levin MJ, Oxman MN, Irwin MR (2008). Sleep disturbance and depression recurrence in community-dwelling older adults: a prospective study. Am J Psychiatry.

[20] Buysse DJ, Angst J, Gamma A, Ajdacic V, Eich D, Rossler W (2008). Prevalence, course, and comorbidity of insomnia and depression in young adults. Sleep.

[21] Kim JM, Stewart R, Kim SW, Yang SJ, Shin IS, Yoon JS (2009). Insomnia, depression, and physical disorders in late life: a 2-year longitudinal community study in Koreans. Sleep.

[22] Szklo-Coxe M, Young T, Peppard PE, Finn LA, Benca RM (2010). Prospective associations of insomnia markers and symptoms with depression. Am J Epidemiol.

[23] Roane BM, Taylor DJ (2008). Adolescent insomnia as a risk factor for early adult depression and substance abuse. Sleep.

[24] Baglioni C, Battagliese G, Feige B, Spiegelhalder K, Nissen C, Voderholzer U, Lombardo C, Riemann D (2011). Insomnia as a predictor of depression: a meta-analytic evaluation of longitudinal epidemiological studies. J Affect Disord.

[25] Stroup DF, Berlin JA, Morton SC, Olkin I, Williamson GD, Rennie D, Moher D, Becker BJ, Sipe TA, Thacker SB (2000). Meta-analysis of observational studies in epidemiology: a proposal for reporting. Meta-analysis Of Observational Studies in Epidemiology (MOOSE) group. JAMA.

[26] Wells G, Shea B, O'Connell D, Peterson J, Welch V, Losos M. The Newcastle Ottawa Scale (NOS) for assessing the quality of nonrandomized studies in meta-analyses. Available at http://www.ohri.ca/programs/clinical_epidemiology/oxford.asp.

[27] Zhang J, Yu KF (1998). What's the relative risk? A method of correcting the odds ratio in cohort studies of common outcomes. JAMA.

[28] Wallace BC, Schmid CH, Lau J, Trikalinos TA (2009). Meta-Analyst: software for meta-analysis of binary, continuous and diagnostic data. BMC Med Res Methodol.

[29] Higgins JP, Thompson SG (2002). Quantifying heterogeneity in a meta-analysis. Stat Med.

[30] Egger M, Davey Smith G, Schneider M, Minder C (1997). Bias in meta-analysis detected by a simple, graphical test. BMJ.

[31] Duval S, Tweedie R (2000). Trim and fill: A simple funnel-plot-based method of testing and adjusting for publication bias in meta-analysis. Biometrics.

[32] Dorheim SK, Bjorvatn B, Eberhard-Gran M (2014). Can insomnia in pregnancy predict postpartum depression? A longitudinal, population-based study. PLoS One.

[33] Brabbins CJ, Dewey ME, Copeland JRM, Davidson IA, McWilliam C, Saunders P, Sharma VK, Sullivan C (1993). Insomnia in the elderly : Prevalence, gender differences and relationships with morbidity and mortality. Int J Geriatr Psychiatry.

[34] Breslau N, Roth T, Rosenthal L, Andreski P (1996). Sleep disturbance and psychiatric disorders: a longitudinal epidemiological study of young adults. Biol Psychiatry.

[35] Foley DJ, Monjan AA, Izmirlian G, Hays JC, Blazer DG (1999). Incidence and remission of insomnia among elderly adults in a biracial cohort. Sleep.

[36] Johnson EO, Chilcoat HD, Breslau N (2000). Trouble sleeping and anxiety/depression in childhood. Psychiatry Res.

[37] Mallon L, Broman JE, Hetta J (2000). Relationship between insomnia, depression, and mortality: a 12-year follow-up of older adults in the community. Int Psychogeriatr.

[38] Roberts RE, Roberts CR, Chen IG (2002). Impact of insomnia on future functioning of adolescents. J Psychosom Res.

[39] Hein S, Bonsignore M, Barkow K, Jessen F, Ptok U, Heun R (2003). Lifetime depressive and somatic symptoms as preclinical markers of late-onset depression. Eur Arch Psychiatry Clin Neurosci.

[40] Perlis ML, Smith LJ, Lyness JM, Matteson SR, Pigeon WR, Jungquist CR, Tu X (2006). Insomnia as a risk factor for onset of depression in the elderly. Behav Sleep Med.

[41] Morphy H, Dunn KM, Lewis M, Boardman HF, Croft PR (2007). Epidemiology of insomnia: a longitudinal study in a UK population. Sleep.

[42] Jansson-Frojmark M, Lindblom K (2008). A bidirectional relationship between anxiety and depression, and insomnia? A prospective study in the general population. J Psychosom Res.

[43] Yokoyama E, Kaneita Y, Saito Y, Uchiyama M, Matsuzaki Y, Tamaki T, Munezawa T, Ohida T (2010). Association between depression and insomnia subtypes: a longitudinal study on the elderly in Japan. Sleep.

[44] Almeida OP, Alfonso H, Yeap BB, Hankey G, Flicker L (2011). Complaints of difficulty to fall asleep increase the risk of depression in later life: the health in men study. J Affect Disord.

[45] Jaussent I, Bouyer J, Ancelin ML, Akbaraly T, Peres K, Ritchie K, Besset A, Dauvilliers Y (2011). Insomnia and daytime sleepiness are risk factors for depressive symptoms in the elderly. Sleep.

[46] Marques M, Bos S, Soares MJ, Maia B, Pereira AT, Valente J, Gomes AA, Macedo A, Azevedo MH (2011). Is insomnia in late pregnancy a risk factor for postpartum depression/depressive symptomatology?. Psychiatry Res.

[47] Thomee S, Harenstam A, Hagberg M (2011). Mobile phone use and stress, sleep disturbances, and symptoms of depression among young adults--a prospective cohort study. BMC Public Health.

[48] Okajima I, Komada Y, Nomura T, Nakashima K, Inoue Y (2012). Insomnia as a risk for depression: a longitudinal epidemiologic study on a Japanese rural cohort. J Clin Psychiatry.

[49] Salo P, Sivertsen B, Oksanen T, Sjosten N, Pentti J, Virtanen M, Kivimaki M, Vahtera J (2012). Insomnia symptoms as a predictor of incident treatment for depression: prospective cohort study of 40,791 men and women. Sleep Med.

[50] Campbell P, Tang N, McBeth J, Lewis M, Main CJ, Croft PR, Morphy H, Dunn KM (2013). The role of sleep problems in the development of depression in those with persistent pain: a prospective cohort study. Sleep.

[51] Gehrman P, Seelig AD, Jacobson IG, Boyko EJ, Hooper TI, Gackstetter GD, Ulmer CS, Smith TC (2013). Predeployment sleep duration and insomnia symptoms as risk factors for new-onset mental health disorders following military deployment. Sleep.

[52] Paudel M, Taylor BC, Ancoli-Israel S, Blackwell T, Maglione JE, Stone K, Redline S, Ensrud KE (2013). Sleep disturbances and risk of depression in older men. Sleep.

[53] Skapinakis P, Rai D, Anagnostopoulos F, Harrison S, Araya R, Lewis G (2013). Sleep disturbances and depressive symptoms: an investigation of their longitudinal association in a representative sample of the UK general population. Psychol Med.

[54] Suh S, Kim H, Yang HC, Cho ER, Lee SK, Shin C (2013). Longitudinal course of depression scores with and without insomnia in non-depressed individuals: a 6-year follow-up longitudinal study in a Korean cohort. Sleep.

[55] Jackson ML, Sztendur EM, Diamond NT, Byles JE, Bruck D (2014). Sleep difficulties and the development of depression and anxiety: a longitudinal study of young Australian women. Arch Womens Ment Health.

[56] Sivertsen B, Lallukka T, Salo P, Pallesen S, Hysing M, Krokstad S, Simon O (2014). Insomnia as a risk factor for ill health: results from the large population-based prospective HUNT Study in Norway. J Sleep Res.

[57] American Psychiatric Association. Diagnostic and Statistical Manual of Mental Disorders, 4th Edition, Text Revision (DSM-IV-TR). American Psychiatric; 2000.

[58] Foley DJ, Monjan A, Simonsick EM, Wallace RB, Blazer DG (1999). Incidence and remission of insomnia among elderly adults: an epidemiologic study of 6,800 persons over three years. Sleep.

[59] Yoo SS, Gujar N, Hu P, Jolesz FA, Walker MP (2007). The human emotional brain without sleep--a prefrontal amygdala disconnect. Curr Biol.

[60] Novati A, Roman V, Cetin T, Hagewoud R, den Boer JA, Luiten PG, Meerlo P (2008). Chronically restricted sleep leads to depression-like changes in neurotransmitter receptor sensitivity and neuroendocrine stress reactivity in rats. Sleep.

[61] Balbo M, Leproult R, Van Cauter E (2010). Impact of sleep and its disturbances on hypothalamo-pituitary-adrenal axis activity. Int J Endocrinol.

[62] Okun ML, Luther JF, Wisniewski SR, Wisner KL (2013). Disturbed sleep and inflammatory cytokines in depressed and nondepressed pregnant women: an exploratory analysis of pregnancy outcomes. Psychosom Med.

[63] Faraut B, Boudjeltia KZ, Vanhamme L, Kerkhofs M (2012). Immune, inflammatory and cardiovascular consequences of sleep restriction and recovery. Sleep Med Rev.

[64] Gimeno D, Kivimaki M, Brunner EJ, Elovainio M, De Vogli R, Steptoe A, Kumari M, Lowe GD, Rumley A, Marmot MG (2009). Associations of C-reactive protein and interleukin-6 with cognitive symptoms of depression: 12-year follow-up of the Whitehall II study. Psychol Med.

[65] Valkanova V, Ebmeier KP, Allan CL (2013). CRP, IL-6 and depression: a systematic review and meta-analysis of longitudinal studies. J Affect Disord.

[66] Gosling JA, Glozier N, Griffiths K, Ritterband L, Thorndike F, Mackinnon A, Hehir KK, Bennett A, Bennett K, Christensen H (2014). The GoodNight study--online CBT for insomnia for the indicated prevention of depression: study protocol for a randomised controlled trial. Trials.

